# Supplementary material for: Complexome profiling of the Chlamydomonas psb28 mutant reveals TEF5 as an early PSII assembly factor
Source: Plant Cell. 2025 Mar 17;37(6):koaf055. doi: 10.1093/plcell/koaf055 (PMC12164590; doi:10.1093/plcell/koaf055)
Supplement: koaf055_Supplementary_Data [file koaf055_supplementary_data.zip › psb28_interactive.zip!/plots/intensity/qualityPlots/peptides/rps2_Cre-1.g2717006.t1.p1.html]

### Cre-1.g2717006.t1.p1

Trivial name: rps2  
SeqCoverage [median]: 10.00  
Mass: 63.16 kDa  
  
 

| Sequence | MissedCleavage | Mass [Da] | wtIntensity | muIntensity | Score |
| --- | --- | --- | --- | --- | --- |
| GAVGLDTR | 0 | 787.42 | 284.05 | 588.00 | 77.06 |
| LGETVQAK | 0 | 844.47 | 1155.51 | 2609.56 | 108.46 |
| LNQVSTSLTQNSFK | 0 | 1565.80 | 0.00 | 347.64 | 123.80 |
| RQELITQSQTLK | 1 | 1443.80 | 308.78 | 1751.50 | 56.12 |
| TNLLNTK | 0 | 802.45 | 0.00 | 546.61 | 84.50 |
|  |  |  | 1748.3 | 5843.3 |  |
